# Nanoparticles of Betalain–Gelatin with Antioxidant Properties by Coaxial Electrospraying: Preparation and Characterization

**DOI:** 10.1021/acsomega.3c04021

**Published:** 2023-10-23

**Authors:** Cielo E. Figueroa-Enriquez, Francisco Rodríguez-Félix, Maribel Plascencia-Jatomea, Armida Sánchez-Escalante, Juan M. Vargas-López, José A. Tapia-Hernández, Dalila F. Canizales-Rodríguez, Daniela D. Castro-Enriquez, Saúl Ruiz-Cruz, Irela Santos-Sauceda, Silvia E. Burruel-Ibarra, José L. Pompa-Ramos

**Affiliations:** † Department of Food Research and Graduate Program, University of Sonora, Hermosillo C.P. 83000, Sonora, Mexico; ‡ Animal Origin Food Technology Coordination, Food and Development Research Center A.C., Hermosillo 83304, Sonora, Mexico; § Department of Polymers and Materials Research, University of Sonora, Hermosillo C.P. 83000, Sonora, Mexico

## Abstract

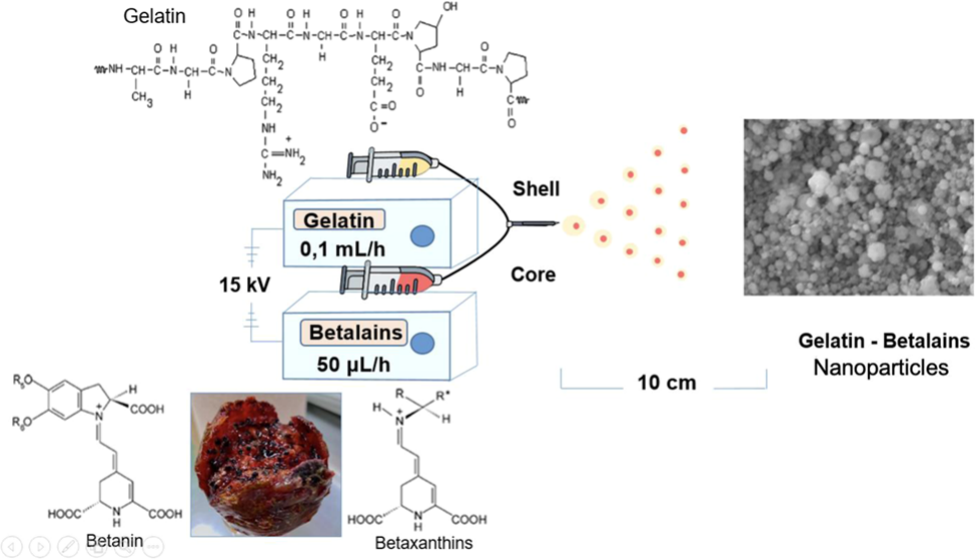

Betalains are bioactive compounds with attractive antioxidant properties for the food industry, endowing them with potential application in food coatings to maintain quality and extend shelf life. However, they have low stability to factors such as light, temperature, and humidity. An alternative to protect bioactive compounds is nanoencapsulation; one of the most used techniques to produce an encapsulation is coaxial electrospraying. In this research, the preparation and characterization of gelatin–betalain nanoparticles were carried out using the coaxial electrospray technique. Betalains were extracted from pitaya (*Stenocereus thurberi*) and encapsulated in gelatin. The obtained material was evaluated by SEM, FTIR, TGA, and DSC techniques and for its antioxidant capacity. By SEM, nanoparticles with spherical and monodisperse morphologies were observed, with betalain concentrations of 1 and 3% w/v and average diameters of 864 and 832 μm, respectively. By FTIR, the interaction between betalain and gelatin was observed through amino groups and hydrogen bonds. Likewise, the antioxidant activity of the betalains was maintained at the time of encapsulation, increasing the antioxidant activity as the concentration increased. The results of the DPPH, ABTS, and total phenols methods were 645.4592 μM T/g, 832.8863 ± 0.0110 μM T/g, and 59.8642 ± 0.0279 mg GAE/g for coaxial nanoparticles with 3% betalains, respectively. Therefore, the coaxial electrospray technique was useful for obtaining nanoparticles with good antioxidant properties, and due to the origin of its components and since the use of toxic solvents is not necessary in the technique, the material obtained can be considered food grade with potential application as a coating on functional foods.

## Introduction

Food quality and safety are the most important factors for the food industry. Therefore, there is interest in replacing synthetic compounds with compounds that come from natural sources, guaranteeing to extend the shelf life of the food, as well as slowing deterioration.^1^ Nanotechnology is used to modify food products to efficiently transport nutrients, vitamins, proteins, and antioxidants to the human body.^2^ Antioxidant compounds from natural sources provide great benefits for health and food safety because they have greater thermal stability and therefore greater antioxidant activity compared to synthetic antioxidants.^3^ Among the antioxidants of natural origin are phenols, ascorbic acid, tocopherols, carotenoids, flavonoids, anthocyanins, and betalains.^4,5^ Each of these compounds comes from different natural sources, such as rosemary, coffee beans, herbs, garlic, peels, fruits, and herbs.^3^ Antioxidant compounds are capable of treating diseases such as cancer and cardiovascular, neurodegenerative, and anti-inflammatory diseases, among others. However, these compounds tend to be sensitive to various factors, such as light, oxygen, heat, humidity, and storage and processing conditions applied to the food. One of the alternatives to control these factors is to employ the use of films, coatings, and encapsulation technologies.^6^

Betalains are pigments found in the fruits, roots, and flowers of plants of the order Caryophyllales. Betalains, in their structure in the presence of betalamic acid with cyclodihydroxyphenylalanil (DOPA cycle), contain nitrogenous compounds, which provide red-violet colors from betacyanins and yellow-orange from betaxanthins. This compound is of great interest due to its high antioxidant activity.^7^ However, its stability is affected by factors such as light, oxygen, water activity, pH, and temperature. Therefore, through encapsulation, it is possible to control these and therefore increase its stability.^8^ The material that is used as a shell and the parameters used are related to the encapsulation efficiency and therefore influence the stability of betalains. This is due to the interactions that the core–shell materials maintain.^9^

Encapsulation is considered one of the most innovative techniques to protect bioactive compounds and be able to deliver them to the site of interest at the right time. Among the most used methods are spray drying, emulsification, nanoprecipitation, solvent evaporation, electrospray, and electrospinning. However, the disadvantages of some of these methods are cost, time, and the use of certain sensitive materials and toxic solvents, as well as the use of high temperatures.^10,11^ Therefore, the advantages of the electrospray technique are highlighted, such as its high encapsulation efficiency and the appearance of biomolecules without the use of high temperatures.^12^

Electrohydrodynamic processes such as electrospraying and electrospinning are considered the most advantageous for the encapsulation of bioactive compounds, such as antioxidant compounds, which are protected within a suitable matrix to easily deposit them in food, in addition to not using high temperatures or toxic solvents.^6,11^ In these processes, the factors that must be controlled in the equipment are voltage, flow rate, the distance between the needle and the manifold, and the diameter of the syringe. In the solution used, the factors that need to be controlled are viscosity, concentration, electrical conductivity, and surface tension, in addition to environmental parameters such as temperature and humidity.^6^ In addition, there are also coaxial electrohydrodynamic techniques that use two different polymeric solutions. Coaxial electrohydrodynamic techniques are a variant of conventional electrohydrodynamic techniques based on placing two syringes with needles of different diameters, each one with a different polymer solution, forming a core–shell structure.^13^ The technique consists of applying a voltage to the polymeric solution, breaking the surface tension, and expelling a jet toward the collector plate. During the flight, the solvent evaporates, and fine spherical droplets are produced, which are nano- or microparticles. This technique is highly efficient for encapsulation.^11^ Different materials used as encapsulants must be studied well to obtain greater protection of the bioactive compound.^14^ Natural polymers have a great advantage as encapsulating materials, since they cause minimal environmental pollution, are inexpensive and nontoxic, and can incorporate antimicrobials, antioxidants, and nutrients. Furthermore, they can be mixed with carbohydrates, proteins, and lipids.^15^ Proteins have been the material of greatest interest due to their unique properties. Gelatin, specifically, is a material used in food to provide better properties of elasticity, stability, and consistency; in addition, it provides excellent barrier properties and permeability and is biocompatible, biodegradable, and nontoxic. Thus, it is possible to incorporate it into coatings or films for food products to extend their useful life.^16^ This protein is derived from collagen, which can be obtained through two processes: an alkaline one, producing type B gelatin, and an acid process, producing type A gelatin. Gelatin can also be extracted from mammals or marine sources.^6^ However, to be used as a material in electrohydrodynamic processes, it is not possible to use water as a solvent, since this would generate gelation. Therefore, solvents such as acetic acid have been implemented in various investigations.^11^

Therefore, the objective of this research is to use the coaxial electrospray method to produce core–shell particles with gelatin and betalains, observe the behavior of different concentrations previously characterized for both gelatin and betalains and varying parameters of the equipment, such as distance and flow, observe the different morphologies and sizes of the particles obtained, and finally characterize the material and study the interactions that have occurred between the components. In addition, the antioxidant capacity of the betalain extract and the particles with and without betalains was also evaluated in order to propose and provide knowledge toward the development of core–shell materials with potential use in food.

## Results and Discussion

### Process for Obtaining Coaxial Nanoparticles

The main factors that must be monitored in the electrospray process are the viscosity of polymeric solutions, density, surface tension, and electrical conductivity, in addition to the conditions of the equipment, such as the flow rate and the distance between the needle and the plate, which allow the solvent used to volatilize and thus give dry particles.^17^ Each of these parameters is related to the size of the particles obtained, so different investigations^18,11^ have revealed that by increasing the gelatin concentration above 11% w/v, fibers are obtained. Therefore, for the objective of this work, such as obtaining particles, 8, 10, and 12% w/v were used as matrix concentrations with 20% v/v acetic acid, the investigation of Gómez^11^ being the starting point for the conditions of these solutions, and the core concentrations were 1, 3, 5, and 7% w/v with 70% v/v ethanol. A flow rate of 0.1 mL/h was used for the matrix and 50 μL/h for the core, since it is a factor that influences the size and shape of the particles.^19^ Solvents such as 20% v/v acetic acid and 70% v/v ethanol were used in order not to generate toxic residues in the material.^20^Table 1 shows the parameters used to obtain the coaxial particles.

**Table 1:** Concentration of the Solutions of Gelatin (Shell) in 20% v/v Acetic Acid and Betalain (Core) in 70% v/v Ethanol, and the Parameters Used in the Coaxial Electrospray Process

concentation solutions		voltage (kV)	flow rate		distance coaxial	
gelatin % w/v	betalain % w/v		gelatin (mL/h)	betalain (μL/h)	level 1 (cm)	level 2 (cm)
10	1	15	0.1	50	10	15
10	3	15	0.1	50	10	15
10	5	15	0.1	50	10	15
10	7	15	0.1	50	10	15

### Characterization of Gelatin Solutions

**Figure 1:**
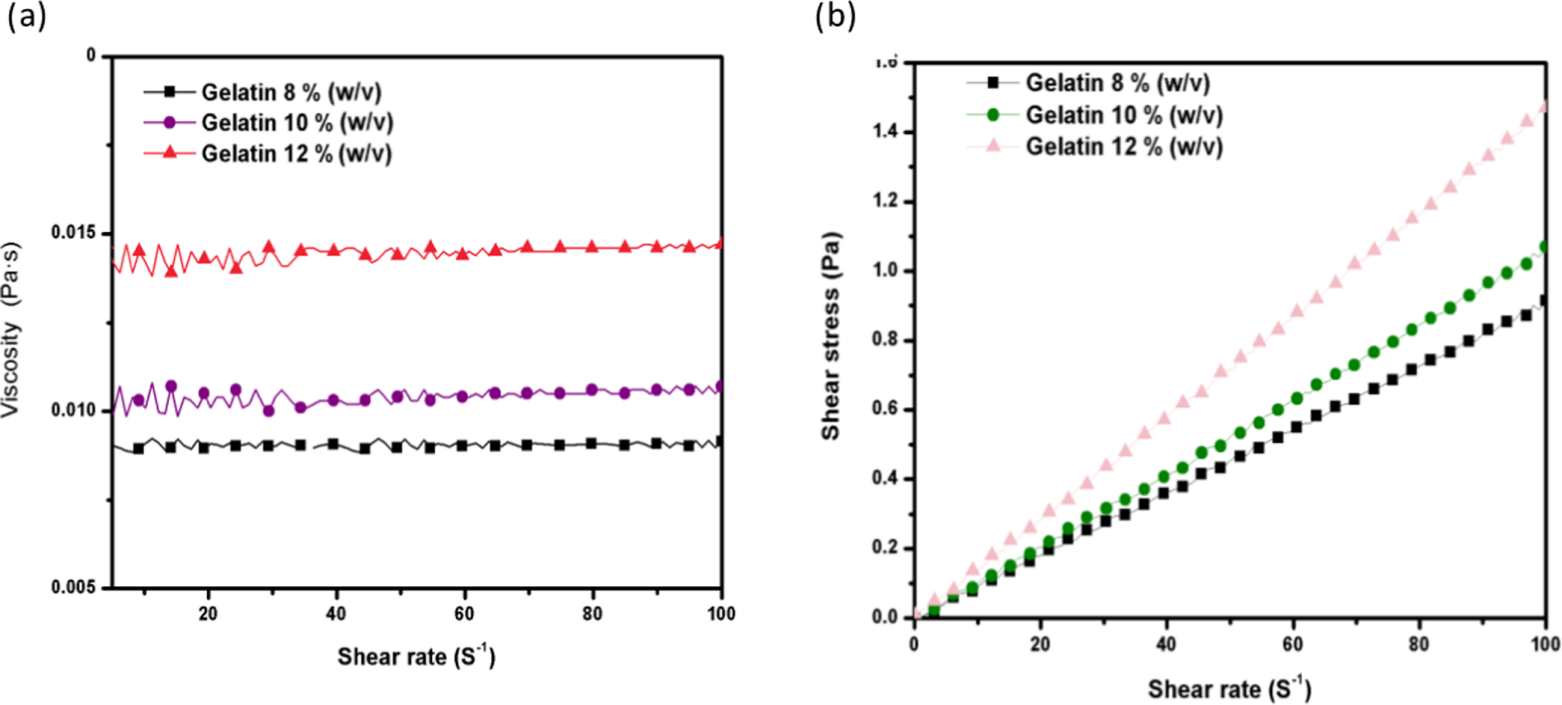
(a) Viscosity flow behavior versus shear stress curves for gelatin solutions at 8, 10, and 12% w/v with acetic acid at 20% v/v. (b) Flow behavior curves of the shear rate versus shear stress of gelatin solutions at different concentrations at 8, 10, and 12% w/v with acetic acid at 20% v/v.

**Figure 2:**
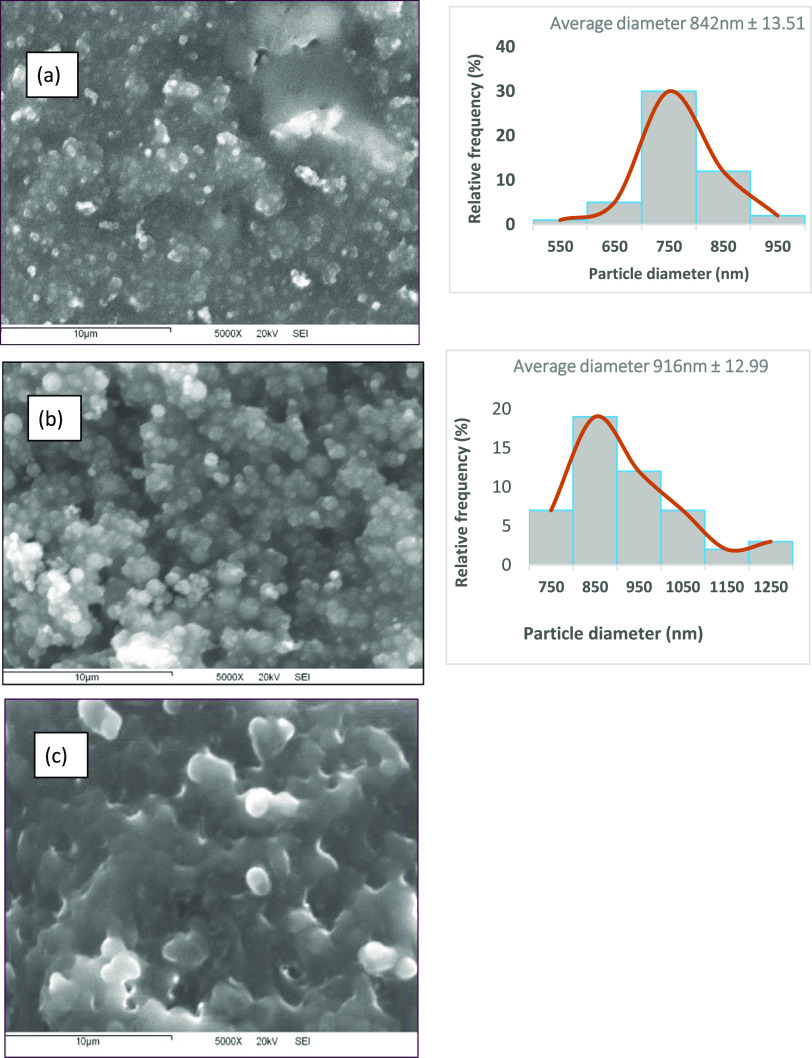
Effect of gelatin concentration on nanoparticle size and morphology: (a) 8%, (b) 10%, and (c) 12% w/v. Conditions: voltage of 15 kV, flow rate of 0.1 mL/h, and collector distance of 10 cm, in conjunction with particle size distribution plots.

The sizes of the particles and their morphology are completely related to the properties of the gelatin solutions,^21^ so each solution was analyzed prior to processing by testing surface tension, viscosity, density, and electric conductivity. Each of the analyses is presented in Table 2. Regarding the electrical conductivity, an increase was shown as the gelatin concentration increased (3.06 ± 0.020, 2.56 ± 0.070, and 3.773 ± 0.005 μs/cm for the electrosprayed solution for 8, 10, and 12% w/v gelatin concentrations, respectively). The differences between all concentrations ranged from 0.50 to 0.71 μs/cm. When passing through the syringe, the liquid acquires a charge, which is defined as the electrical conductivity that the solution acquires. A solution with a higher electrical conductivity gets quickly charged, which results in a stable cone jet mode with smaller and uniformly sized particles. However, when a solution with a low electrical conductivity value is used, a small amount of charge or no charge is acquired, which results in an unstable cone jet mode, forming larger particles with a large size variation.^22,23^ In the present study, the solution with the lowest gelatin concentration (12% w/v) had a high electrical conductivity (3.773 ± 0.005 μs/cm), which is related to nonuniform sizes and morphologies. The electrical conductivity value depends on the type and concentration of the solvent in the solution.^24^ Regarding the results of surface tension, a behavior inversely proportional to the concentration of the polymeric solutions was observed, providing information on the behavior of the jet when it breaks down into fine droplets, affecting the shape of the particles due to surface tension.^25^Table 2 presents the results obtained, where the surface tension values of gelatin solutions with different concentrations 8, 10, and 12% w/v are 43.27 ± 0.43, 42.61 ± 0.70, and 41.43 ± 0.55 mN/m, respectively. The differences between all concentrations ranged from 0.66 to 0.84 mN/m. However, the different morphologies shown in the SEM micrographs in Figure 2can be attributed, especially, to the properties of viscosity and density. The rheological data of the solutions obtained through the analysis of shear rate versus shear stress (Figure 1) from 0 to 100 s^–1^. It was possible to apply the power law model (Table 3), where for a gelatin concentration of 10% w/v, an n value of around 1 and an R^2^ of 0.99 were presented. These results are related to a Newtonian-type fluid, which tells us that the viscosity remains constant with respect to the change in speed increase cutoff. These results coincide with refs (26,11), where different solutions of gelatin (8, 10, and 20% w/v) and 20% v/v acetic acid presented the same behavior. Therefore, the density of the solutions also increased as the concentration of the polymer solution increased (1.0576 ± 0.0034, 1.0826 ± 0.0063, 1.1201 ± 0.0007 g/cm^3^ for 8, 10, and 12% w/v gelatin, respectively). The difference between the solutions was 0.062 g/cm^3^. When a low shear rate occurs in the 8 and 10% w/v gelatin solutions, the apparent viscosity is significantly different from that of the 12% w/v gelatin solution. Therefore, when solutions with lower concentrations are subjected to a shear cut, weak bonds begin to break, these being the intramolecular network. However, once this is achieved, the shear cutting behavior remains constant.^27^ This property has a crucial impact on the development of a Taylor cone in the electrospray process. At a high density, the Taylor cone tends to form large particles, while at a lower density, the Taylor cone changes to a stable cone jet mode or multiple cone jet mode with smaller particles.^28^

**Table 2:** Physicochemical Characterization of Gelatin Solutions: Density, Electrical Conductivity, and Surface Tension

concentration (%)	density g/cm^3^	conductivity μS/cm	surface tension mN/m
8	1.05 ± 0.02^c^	3.06 ± 0.41^b^	43.27 ± 0.43^a^
10	1.08 ± 0.01^b^	3.68 ± 0.05^a^	42.61 ± 0.70^a^
12	1.12 ± 0.04^a^	3.77 ± 0.00^a^	41.43 ± 0.55^b^

Note: The values correspond to the mean ± standard deviation. Different letters (a, b, c) between treatments are significantly different if *p* < 0.05.

**Table 3:** Parameters of the Power Law of the Gelatin Solution at Different Concentrations Using 20% v/v Acetic Acid as the Solvent

concentration of gelatin (% w/v)	*K* (Pas^n^)	*n*	*R*^2^
8	0.0145 ± 0.0013	0.9643 ± 0.2743	0.9909 ± 0.0210
10	0.0131 ± 0.0052	0.9931 ± 0.0904	0.9944 ± 0.0050
12	0.0186 ± 0.0046	0.9459 ± 0.0421	0.9940 ± 0.0046

### Scanning Electron Microscopy

Gelatin nanoparticles were obtained by the electrospraying technique and analyzed by SEM. Figure 2 shows the micrographs of each concentration of gelatin (8, 10, and 12% w/v) with 20% v/v acetic acid, and Table 4 shows the results of the average diameters obtained. In the micrograph of 8% w/v gelatin, dispersed particles of nonuniform size with a mean diameter of 842 nm ± 13.5 were observed. It was also observed that this concentration (8% w/v gelatin) was not sufficient to form nanoparticles with a completely spherical morphology. In the micrograph of 10% w/v gelatin, uniform and monodisperse particles with a more spherical morphology were observed, without the formation of sheets or fibrils and with a minimum increase in size and a mean diameter of 916 nm ± 12.99, showing a PDI value of 0.014 (Table 4). On the other hand, in the micrograph with a higher gelatin concentration of (12% w/v), few particles with sheet formation were observed; therefore, diameter measurement was not performed. The presence of chain cross-links for a gelatin concentration of 12% w/v explains the production of spun fibrils instead of powdered particles.^29^ The differences in the morphology of each material are mainly attributed to changes in the physicochemical properties of the solutions. The results obtained are consistent in gelatin–acetic acid systems using type B bovine skin gelatin.^26^

**Table 4:** Average Diameter and Polydispersity Index of Gelatin Particles (8 and 10% w/v)

concentration of gelatin (w/v)	diameter (nm)	PDI
8%	842.0 ± 13.5^b^	0.016
10%	916 ± 12.99^a^	0.014

Note: Values correspond to mean ± standard deviation.

Different letters (a, b) between treatments are significantly different if *p* < 0.05.

### Scanning Electron Microscopy of Coaxial Nanoparticles

**Figure 3:**
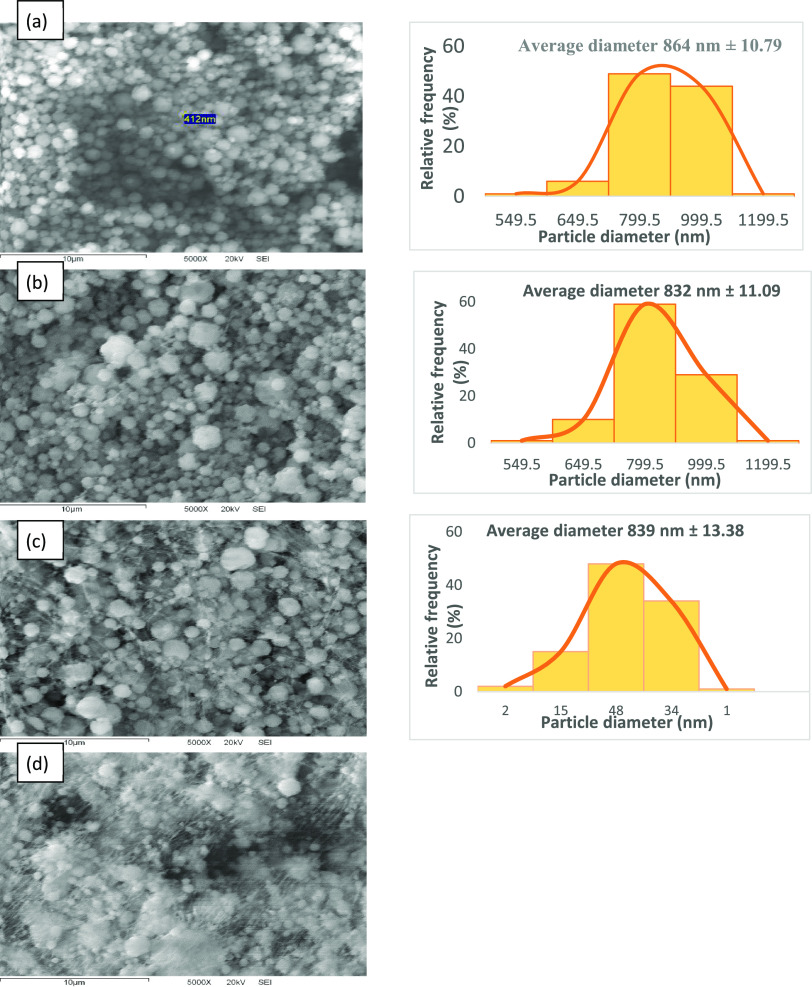
Micrographs of coaxial nanoparticles obtained with 10% w/v gelatin with 1% (a), 3% (b), 5% (c), and 7% (d) w/v betalains at 5,000 magnifications. Coaxial electrospray conditions: voltage of 15 kV, shell flux of 0.1 mL/h, core flux of 50 μL/h, and needle–plate distance of 10 cm, in conjunction with the particle size distribution graphs.

The coaxial gelatin–betalain nanoparticles were characterized by SEM analysis. SEM micrographs of the coaxial nanoparticles were obtained with a gelatin concentration of 10% w/v in the shell and concentrations of betalains of 1, 3, 5, and 7% w/v in the core (Figure 3). The mean diameters of the coaxial nanoparticles were 864, 832, and 839 nm for 10% w/v gelatin and 1, 3, and 5% w/v betalains, respectively, observing that these values are lower than those presented by gelatin particles without betalains. Therefore, in the electrospray technique, the morphology of the particles corresponds to the type of solvent used, while the size of the particle depends merely on the flow rate used.^30^ Likewise, the viscosity and aggregation of a bioactive agent play important roles in the size of the particles, since the stability of the jet of the compound solution can define and control the morphology, as well as the size of the particles. Therefore, in this technique, to obtain even more compact particles, it would be necessary to use solutions of lower viscosity.^31^ Soto-Cruz^32^ mentioned that this could occur due to chain packing through molecular interactions when adding the bioactive compound. In these three cases mentioned, values of the polydispersity index very close to 0 were obtained, indicating a morphology with a tendency toward monodispersity (Table 5). In the case of the coaxial nanoparticles of 10% w/v gelatin and 7% w/v betalains, in which a higher concentration of betalains was used, it was not possible to obtain the diameter value, since they had fibrils, and therefore nor the polydispersity index value. These results have been consistent with studies performed on bovine skin type B gelatin systems and bioactive compounds in the core of the material, as there is an entanglement of peptide chains and chain–chain interactions, leading to fibril formation.^26,11^ The most spherical morphology, free of fibrils as residues, was presented by the lowest concentrations of betalains of 1 and 3% w/v.

**Table 5:** Mean Diameter and Polydispersity Index of Coaxial Gelatin–Betalain Particles, with Gelatin (Shell) at 10% w/v and Betalains (Core) at Different Concentrations

concentration of betalains (% w/v)	diameter (nm)	PDI
1	846 ± 10.79^a^	0.012
3	832 ± 11.09^a^	0.013
5	839 ± 11.38^a^	0.013

Note: Superscript letters are used to indicate which values are significantly different, and in this case, there is no difference between the values obtained.

### Fourier Transform Infrared Spectroscopy (FTIR)

**Figure 4:**
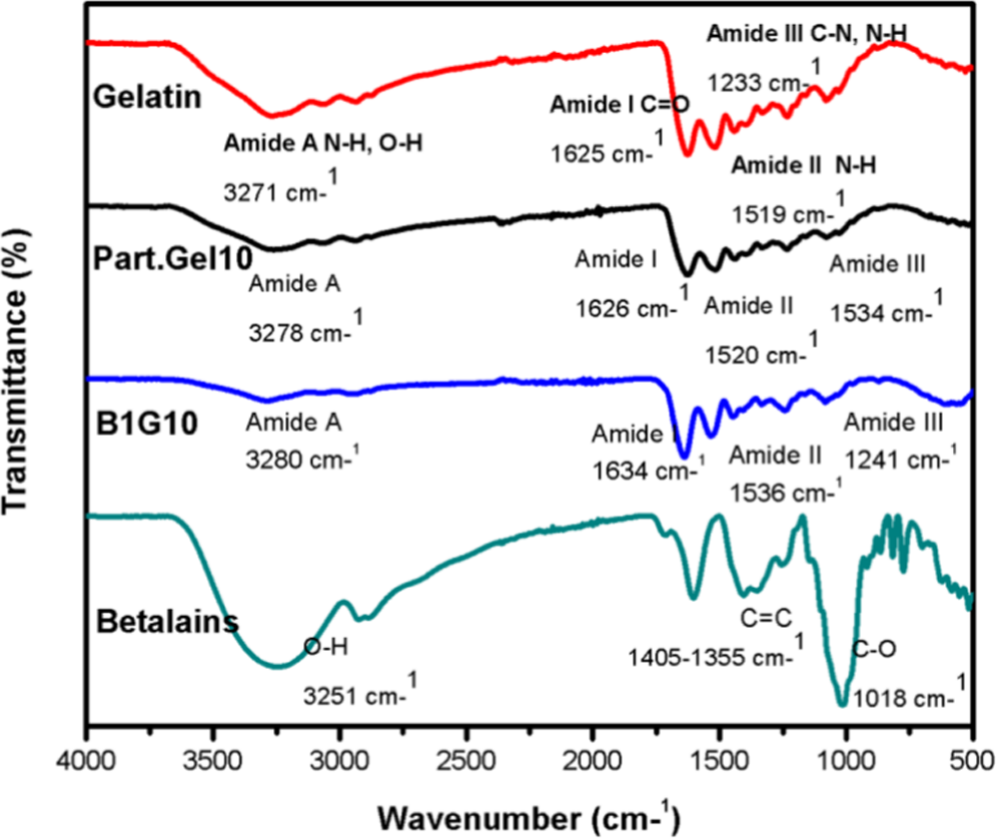
FTIR spectrum of gelatin, 10% w/v gelatin nanoparticles (Part.Gel.10), 1% w/v betalain coaxial nanoparticles with 10% w/v gelatin, and pure betalains (betalains).

To confirm the incorporation of betalains into gelatin nanoparticles, Fourier transform infrared spectroscopy (FTIR) using an ATR accessory was used. The spectra of the starting compounds (gelatin and betalains), uncharged gelatin particles, and a sample of 10% w/v coaxial gelatin particles with 1% w/v betalains were analyzed (Figure 4). Gelatin presents three typical amide bands characteristic of proteins.^33^ Initially, amide band A is presented at 3271 cm^–1^, corresponding to the amino and hydroxyl groups. The following representative bands are amide I at 1625 cm^–1^, which corresponds to the vibrations of the carbonyl bond (C=O), the amide II band is present at 1519 cm^–1^, corresponding to N–H bending and C–N stretching, and the amide III band appears at 1233 cm^–1^, corresponding to the N–H bending. The spectra of uncharged gelatin nanoparticles and coaxial nanoparticles followed the same pattern of bands characteristic of gelatin. Stretching vibrations were presented for the N–H and O–H bonds of the amide A band (3278 cm^–1^ for uncharged gelatin nanoparticles and 3280 cm^–1^ for coaxial nanoparticles), stretching of the amide I band of the C–O bond (1626 cm^–1^ for uncharged gelatin nanoparticles and 1634 cm^–1^ for coaxial nanoparticles), amide II N–H bond bending (1520 cm^–1^ for uncharged nanoparticles and 1536 cm^–1^ for coaxial nanoparticles), and bending for the N–H bond (1534 cm^–1^ for uncharged nanoparticles and 1241 cm^–1^ for coaxial nanoparticles). In the spectra of the starting compounds described above, a shift in amide A from 3271 to 3280 cm^–1^ is shown, attributed to the amino and hydroxyl groups, confirming the interaction between gelatin and betalains, given by the amino groups coupled to the bonds of hydrogen from gelatin.^34^ Likewise, the shift observed in amide III from 1233 to 1241 cm^–1^ is attributed to the conformational change in the protein.^11^ The spectrum of the betalains presented a band at 3251 cm^–1^, corresponding to the tension of the −OH group due to the presence of phenols, and at 1405–1355 cm^–1^, corresponding to the stretching of the aromatic ring. The intense band at 1018 cm^–1^ is characteristic of this type of pigment, showing the deformation of the aromatic ring of betalains.^35^

**Figure 5:**
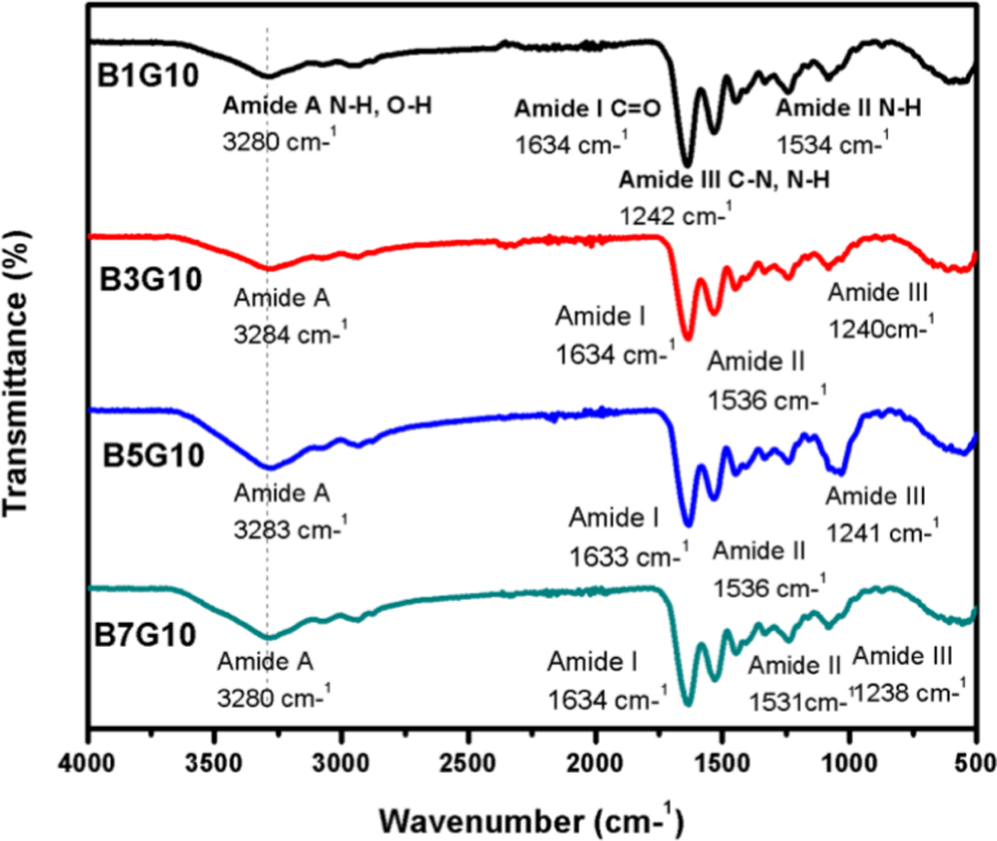
FTIR spectrum of coaxial particles: 1% w/v betalains with 10% w/v gelatin (B1G10), 3% w/v betalains with 10% w/v gelatin (B3G10), 5% w/v betalains with 10% w/v gelatin (B5G10), and 7% w/v betalains with 10% w/v gelatin (B7G10).

Likewise, the characterization of the coaxial gelatin nanoparticles at 10% w/v and betalains at 1, 3, 5, and 7% w/v, named B1G10, B3G10, B5G10, and B7G10, respectively, was carried out. The corresponding spectrum (Figure 5) shows the same pattern for all of the coaxial nanoparticle samples. However, different intensities are observed in the bands, highlighting the characteristic amide A band. When the concentration of betalains increases, the band becomes smaller and shows more intensity, which is due to the amino and hydroxyl groups present in the compound having interactions between hydrogen bonds and amino groups.^36^ In amide bands I and II, no significant shifts were observed. However, for the band corresponding to amide III, a decrease in wavenumber from 1242 to 1238 cm^–1^ was observed, which is related to a variation of hydrogen bonds due to the concentration of betalains.^37^

## Thermal Analysis

### Thermogravimetric Analysis

**Figure 6:**
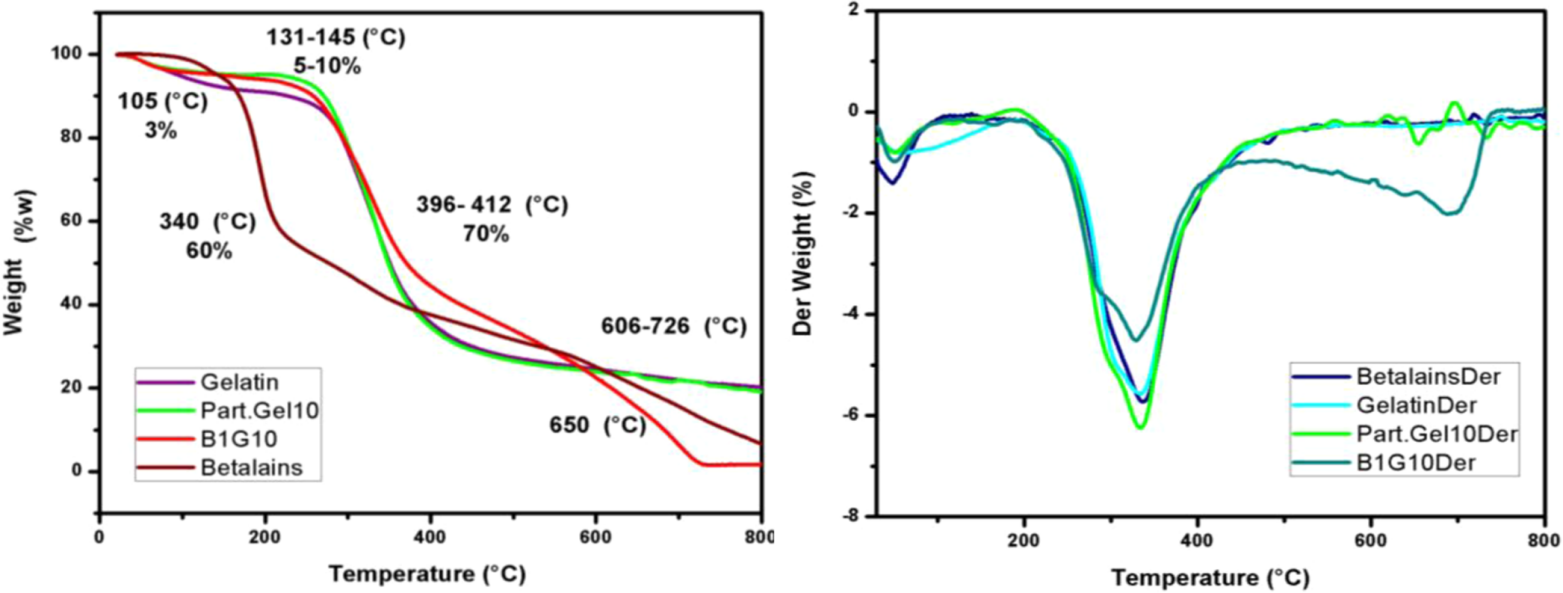
Thermograms of TGA and derivative of controls: gelatin, betalains, betalain–gelatin coaxial particles (B1G10), and gelatin particle (Part.Gel10).

**Figure 7:**
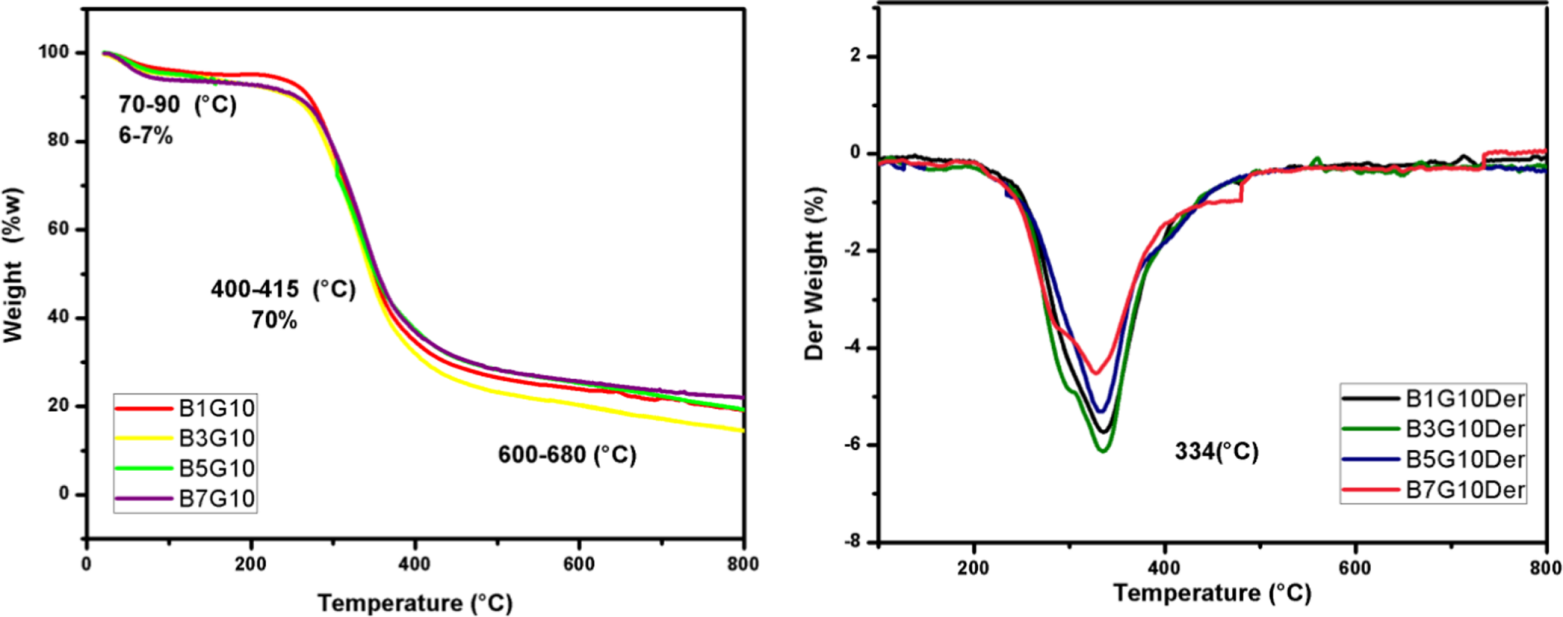
Thermograms of TGA and derived from samples: betalain–gelatin coaxial particles (B1G10), (B3G10), (B5G10), and (B7G10).

Thermogravimetric analysis (TGA) and the first derivative (DTG) analysis of the starting compounds and the coaxial nanoparticles were performed to study the degradation behaviors with respect to temperature. The thermograms of the betalains, gelatin, 10% w/v coaxial gelatin particles with 1% w/v betalains, and uncharged gelatin particles showed three different stages in the loss curve of weight (Figure 6). The first stage was observed in a range from 131 to 145 °C (10–5% weight loss), which is related to the loss of adsorbed and bound water, especially in the samples containing gelatin due to its hygroscopic character.^11^ The second stage, which corresponds to the greatest weight loss, is in a range of 396–412 °C (70% weight loss) and is associated with the breaking of protein chains and the breaking of peptide bonds.^38^ Finally, the third stage is between 606 and 726 °C, associated with the thermal decomposition of the gelatin networks.^39^ In the case of betalains, a small stage is present at 105 °C, which corresponds to 3% of its weight and is attributed to the decomposition of betalains by dehydroxylation and the separation of betalamic acid from the entire molecule.^35^ Furthermore, as shown in Figure 6, DTG can confirm that the starting compounds show lower thermal stability compared to the blank of the coaxial nanoparticles, showing that the interaction between gelatin and betalains increases the thermal stability. The thermograms of the coaxial nanoparticles (Figure 7) show the same behavior with the three stages of weight loss. The first stage is shown in a range of 70–90 °C, which is attributed to the loss of water. In the second stage, there is a degradation of 70% of its weight, in a range of 400–415 °C, and finally, the third stage is shown at 660–680 °C. The specific values that correspond to the three stages of weight loss of each sample are indicated in Table 6. When studying the thermograms, it can be concluded that since the betalains are encapsulated, they have greater thermal stability, since the carboxyl group of the gelatin reacts with the amino group of the betalains, causing a change in the side chain of the protein and protecting it from thermal degradation.^11^ Amani et al.^40^ obtained similar results, where they encapsulated rosemary oil in a gelatin complex, noting that this complex improved the thermal stability of the bioactive compound. By confirming the above with the DTG (Figure 7), it is observed that the coaxial nanoparticles with 3% w/v betalains are the ones that presented greater stability at 334 °C.

**Table 6:** Thermal Stability Obtained by TGA: Betalain–Gelatin Coaxial Particles ((B1G10), (B3G10), (B5G10), and (B7G10))

	first stage		second stage		third stage	
sample	temperature (°C)	weight loss (%)	temperature (°C)	weight loss (%)	temperature (°C)	weight loss (%)
gelatin	145	10	412	70	606	>100
Part.Gel10	131	5	396	65	618	>100
B1G10	133	7	403	68	726	>100
betalains	105	3	340	60	650	>100
B1G10	70	6	400	69	600	>100
B3G10	78	6	403	69	616	>100
B5G10	86	7	410	70	646	>100
B7G10	90	7	415	70	680	>100

### Differential Scanning Calorimetry

**Figure 8:**
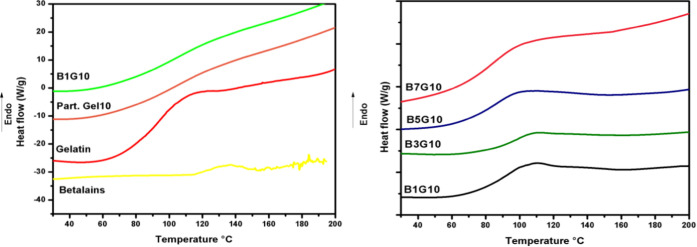
DSC of (a) samples: gelatin, 10% w/v gelatin nanoparticles (Part.Gel.10), 1% w/v betalain coaxial nanoparticles with 10% w/v gelatin, and pure betalains (betalains) and (b) coaxial particles: 1% w/v betalains with 10% w/v gelatin (B1G10), 3% w/v betalains with 10% w/v gelatin (B3G10), 5% w/v betalains with 10% w/v gelatin (B5G10), and 7% w/v betalains with 10% w/v gelatin (B7G10).

Using the differential scanning calorimetry technique, the encapsulation of a bioactive compound within a material as a shell can be confirmed by comparing the thermograms of the nonencapsulated compound and the encapsulated sample under heating conditions.^41^Figure 8 shows the thermograms of the control samples presented as pure betalains, pure gelatin, 10% w/v gelatin particles without loading, and coaxial particles with 1% w/v betalains and 10% w/v gelatin. Figure 8 shows the thermograms of the coaxial particles with the variations of betalains (1, 3, 5, and 7% w/v) encapsulated with 10% w/v gelatin. The thermograms of these last samples revealed the same pattern. In the thermogram where the pure gelatin is presented, a value of 88.29 °C was observed, which corresponds to a glass transition (Tg); also, at 112.48 °C, a peak corresponding to an endothermic transition (Tg) was presented. The glass transition temperature is related to the transition from glass to rubber, corresponding to the amino acids of the peptide chain of gelatin. Likewise, when the bioactive compound is added, rigidity in the movement of the amorphous polymer chain and greater hydrogen bonds can be observed.^42^ Mukherjee^43^ reported very similar values where a first-order glass transition was observed at a temperature range of 80–90 °C and a second-order endothermic transition was observed in a temperature range of 110–115 °C. In the case of the betalain extract, a pronounced endothermic peak was observed at 136 °C, indicating the melting point. Mohammed^44^ presented a similar result with an endothermic melting peak at around 103 °C corresponding to the extract containing nonencapsulated betalains. However, this peak was not observed in the thermograms corresponding to the coaxial nanoparticles (Figure 8), which means that gelatin as an encapsulating agent confers greater thermal stability to betalains. Mourtzinos^41^ mentioned that the behaviors analyzed in the thermograms can indirectly evidence the encapsulation of the bioactive compound. Otálora^45^ mentioned that the interactions between polymers and betalains through hydrogen bonding could cause the modification of the glass transition temperature of the polymers.

### Antioxidant Activity

The results obtained for the antioxidant activity of the coaxial nanoparticles of 1 and 3% w/v betalains encapsulated with 10% w/v gelatin (B1G10 and B3G10, respectively) are shown in Table 7, and in addition, uncharged gelatin particles and the ultrafiltered betalain pure extract were also analyzed. The DPPH, ABTS, and total phenols methods were used. In the results, it is observed that the ultrafiltered extract of betalains has a high antioxidant capacity. Similar results were obtained in studies by refs (35,7), where they analyzed the extract of ultra- and non-ultrafiltered betalains, demonstrating that there was a significant difference between an extract without ultrafiltration and an ultrafiltrate. The ultrafiltrate gave a positive response because the extract might contain pro-oxidant compounds that were eliminated when it was subjected to the ultrafiltration process.^7^ Therefore, in this investigation, the extract used was ultrafiltered, which gave values of 1130.6211 ± 0.0180 mg GAE/g for ABTS, inhibiting 62.47%, 1617.1169 ± 0.003 (μM T/g) for DPPH, inhibiting 80.63%, and 75.85 ± 0.0386 mg GAE/g for total phenols. However, the results show that the addition of betalains to the gelatin particles improved the activity of neutralizing DPPH and ABTS radicals. Betalains maintain their stability and therefore their antioxidant activity, related to the ability to donate electrons from the −OH groups in their phenolic structure, when interacting with gelatin and being encapsulated, presenting an increase in antioxidant activity due to the content of cyclic amine and phenolic groups that are electron donors, as well as hydroxyl groups.^46^ On the other hand, phenolic compounds are considered an important group of phytochemicals. These are found in the pulp and skin of pitaya.^47^ The results of total phenols that were obtained in the betalains of the pitaya *Stenocereus thurberi* extract (70.6161 mg GAE/g) are above those obtained for other species of pitaya. Quiroz^48^ presented values of 13.6 mg GAE/g for pitaya *Stenocereus stellatus*, 0.53 mg GAE/g for *Streptomyces griseus*, and 5.79 mg GAE/g for *S. stellatus*. However, Wu^49^ reported a value of 42.4 mg GAE/g for the species *Hylocereus spp*. in the total phenols test, a value similar to that obtained in this investigation. The results of the antioxidant capacity of the B1G10 nanoparticles were 758.049 ± 0.0334 μM T/g by ABTS, 499.9050 ± 0.0180 μM T/g by DPPH, and finally 68.744 ± 0.0540 mg GAE/g by total phenols. For the B3G10 nanoparticles, the values obtained were 832.8863 μM T/g by the antiradical ABTS, 645.4592 μM T/g by DPPH, and 59.8642 mg GAE/g by total phenols. The uncharged gelatin particles were analyzed using the same methods; the results obtained showed good antioxidant activity, especially against the antiradical ABTS, with a value of 630.2143 μM T/g. The difference between the coaxial particles with different concentrations of betalains is due to the fact that increasing the concentration of betalains increases the antioxidant capacity. The same thing happened in the total phenols test, being able to trap free radicals in addition to reducing metal ions.^33^ Also, it is important to consider that the presence or position of the hydroxyl groups of the molecule increases the antioxidant capacity.^50^ Gelatin, as an encapsulating compound, conferred stability to betalains. In addition, they continue to present antioxidant activity due to interactions between functional groups or molecular structures and even because of the ramifications that can help the stability of the extracts.^51^

**Table 7:** Values of Antioxidant Capacity by DPPH, ABTS, and Total Phenols for the Pure Extract of Betalains, Coaxial Particles with 1 and 3% w/v Betalains with 10% w/v Gelatin (B1G10 and B3G10, Respectively), and Uncharged Gelatin Particles (Gelatin Particles 10)

	DPPH		ABTS		
sample	(μM T/g)	% inhibition	(μM T/g)	% inhibition	total phenols mg GAE/g
B1G10	499.9050 ± 0.01^a^	67.727 ± 0.03^a^	758.0495 ± 0.03^a^	23.633 ± 0.02^a^	68.744 ± 0.05^ab^
B3G10	645.4592 ± 0.00^a^	77.637 ± 0.05^a^	832.8863 ± 0.01^a^	22.338 ± 0.05^a^	59.8642 ± 0.02^ab^
gelatin particles 10	471.2464 ± 0.02^c^	55.202 ± 0.05^c^	630.2144 ± 0.16^b^	50.612 ± 0.04^b^	
betalain extract	1617.1169 ± 0.00^c^	80.636 ± 0.00^c^	1130.6211 ± 0.01^c^	62.482 ± 0.85^c^	75.8569 ± 0.03^a^

Note: Superscript letters are used to indicate which values are significantly different.

## Experimental Section

### Materials and Reagents

Type B gelatin from bovine skin, with a reported gel concentration of 175 g of bloom, was obtained from Sigma-Aldrich. Glacial acetic acid (J.T. Baker, EU), 99% v/v ethanol (Meyer, MX), 2,2-diphenyl-1-picrylhydrazyl (DPPH, Sigma-Aldrich, EU), 2,2-azino-bis (3-ethylbenzothiazoline-6-sulfonic) (ABTS, Sigma-Aldrich, CA), and Folin Ciocalteu (Sigma-Aldrich, EU) were used.

### Obtaining Betalain Extract

Pitayas (*S. thurberi*) were collected in the municipality of Carbó, which is located in the west of the state of Sonora, México, between the geographic coordinates 29°41′ north latitude and 110°57′ west longitude. The pitayas were transported to the agrifood nanotechnology laboratory of the University of Sonora to carry out the extraction. The extraction was performed by ultrasonication based on the technique proposed by Castro,^35^ where 2 g of seedless pulp was taken to be macerated with 34 mL of distilled water. Once this mixture was obtained, it was kept in a water bath to be sonicated for 27 min (Branson, M3800H, Mexico City, MX) and then agitated for 20 min using a horizontal stirrer (VWR, mini blot mixer), followed by being placed in a centrifuge at 5000 rpm for 10 min (Eppendorf, 5804 R, Hamburg, DE). All extractions were performed in the dark. Once the supernatant was obtained, ultrafiltration was carried out using a 50 mL Amicon cell (Millipore, Model 8050, Darmstadt, DE) and placing membranes (Millipore, Temecula) with a molecular weight cutoff of 1 kDa at 25 °C and applying a pressure of 50 psi of nitrogen gas. Once the ultrafiltered extract was obtained, it was lyophilized. The extract was then stored in amber containers to proceed with the analyses.

### Preparation of Gelatin Solutions

Aqueous gelatin solutions of different concentrations, such as 8, 10, and 12% w/v, were prepared by dissolving the biopolymer in 20% v/v acetic acid at 30 °C under magnetic stirring.

### Preparation of Betalain Solution

Aqueous solutions of the betalain extract of different concentrations, namely, 1, 3, 5, and 7% w/v, were prepared by dissolving the extract in 70% v/v ethanol at 25 °C under magnetic stirring.

### Characterization of the Solutions

#### Viscosity

The viscosity analysis was carried out using the methodology proposed by Bhushani^52^ with modifications. Measurements were performed on a modular compact rheometer (MCR, Anton Paar, Germany) using a concentric cylinder geometry. The analysis was performed for the gelatin solutions at different concentrations (8, 10, and 12% w/v). Conditions included a shear rate from 0 to 100 s^–1^ at 25 °C. Determinations were made in triplicate. Once the data were obtained, the power law was used.

#### Density

Density was calculated using the pycnometer method described by Tapia.^53^ First, an empty pycnometer was brought to constant weight, and the measurement was classified as M1. Subsequently, the pycnometer was taken to add water, which was called M2. Afterward, the gelatin solutions were placed in the pycnometer. Subsequently, they were weighed, and this measurement was denominated as M3. The density of gelatin solutions was determined based on eq 1. Determinations were performed in triplicate.
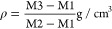
1

#### Electrical Conductivity

Electrical conductivity was determined using the methodology described by Prietto.^54^ A Hanna Instruments conductivity meter (Model HI 2550; pH, ORP, and EC/TDS/NaCl meter) was used. Determinations were performed in triplicate.

#### Surface Tension

The surface tension was measured by the placed drop method, where an IT Concepts tensiometer was used. Measurements were performed in triplicate.

#### Parameters Applied in Coaxial Electrospray

The different solutions of both gelatin and betalains were processed using coaxial electrospray equipment using two pumps, a Tongli Tech TL-F6 for the shell and a KD Scientific, Holliston, MA for the core, using a high-voltage source of 15 kV. Each solution was placed in a 10 mL syringe with a diameter of 15 mm using flow rates of 0.1 mL/h for the core and 50 μL/h for the core. The distance between the needle and the plate varied between 10 and 15 cm. Finally, the material obtained was recovered on an aluminum plate. Table 8 shows the parameters used in the processing to obtain the gelatin-based material.

**Table 8:** Concentrations and Parameters of the Gelatin Solutions Used

sample	concentration (% w/v)	flow (mL/h)	voltage (kV)	distance (cm)
gelatin	12	0.1	15	10
gelatin	10	0.1	15	10
gelatin	8	0.1	15	10

### Particle Characterization

#### Scanning Electron Microscopy (SEM)

The morphological characteristics of the particles were studied using a scanning electron microscope (JEOL JSM-5410LV, Tokyo, Japan) equipped with an INCA system and an energy-dispersive X-ray (EDS) microanalysis detector (Oxford Instruments), operating with an accelerating voltage of 20 kV and an amplification of 10000×. The analysis process of the sample was performed by using a secondary electron detector. Moreover, the polydispersity index (PDI) of the electrospun nanofiber was obtained by eq 2
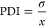
2where σ represents the standard deviation and *x* represents the average diameter of the nanoparticles. PDI close to 0 represents monodispersed particles, and PDI close to 1 represents polydispersed particles.

#### Fourier Transform Infrared (FTIR) Analysis

The particles and starting compounds, such as gelatin, betalain extract, uncharged gelatin particles, and coaxial gelatin–betalain nanoparticles, were studied by Fourier transform infrared spectroscopy (FTIR) using a PerkinElmer Frontier spectrometer with an ATR attachment using a scale of 4000–400 cm^–1^ of transmittance to observe the possible chemical–structural interactions between the components of the material.

#### Thermogravimetric Analysis

Degradation analysis was performed with respect to the temperature of coaxial gelatin–betalain nanoparticles as well as starting compounds such as gelatin, betalain extract, and uncharged gelatin particles. It was analyzed by thermogravimetric analysis (TGA) and the derivative of weight loss (TDG) using a PerkinElmer equipment model: Pyris 1. Samples of approximately 5 mg were taken and subsequently heated to 600 °C, with a heating rate of 10 °C/min, under a nitrogen gas flow of 20 mL/min.

#### Differential Scanning Calorimetry (DSC)

To determine the phase changes of gelatin nanoparticles, coaxial gelatin–betalain nanoparticles, and pure gelatin–betalain compounds with respect to temperature, the differential scanning calorimetry (DSC) technique was used, using a conventional PerkinElmer DSC equipment model 8500. Approximately 7 mg of the sample was taken and sealed to be heated at 10 °C/min from 25 to 200 °C under a nitrogen flow of 40 mL/min.

#### Antioxidant Activity

The ABTS, DPPH, and total phenols tests were performed to evaluate the antioxidant capacity of the coaxial particles, the uncharged gelatin particles, and the betalain extract.

#### ABTS

For the ABTS assay, the methodology proposed by Re^55^ was used, where 270 μL of the radical solution was first taken, which contained an ethanol concentration of 0.043 mg/mL; then, it was mixed with 20 μL of the sample, leaving it at rest for 30 min in the dark. Absorbance was measured at 734 nm in a microplate reader (Thermo Scientific, Multiskan GO, FI). The results were expressed for both radicals in mM ET/g and in % inhibition according to eq 3
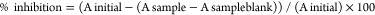
3where A initial is the reagent + H_2_O, A sample is the reagent + sample, and A sample blank is H_2_O + sample.

#### DPPH

Based on the methodology proposed by Pérez-Perez,^56^ determination of the antioxidant activity by DPPH was carried out, where first, a solution of the DPPH radical was prepared by adding 1.5 g of the DPPH radical to 50 mL of methanol, proceeding to adjust the solution to an absorbance of 0.7 ± 0.01 at 515 nm. 200 μL of the radicals were added to 20 μL of the sample, mixed, and allowed to stand for 30 min in the dark, and the absorbance was measured at 515 nm.

#### Total Phenols

The analysis of total phenols was carried out using the previous methodology.^57^ 10 μL of each sample were taken to mix with 25 μL of FC 1N and then left to stand for 5 min. Next, 25 μL of 20% Na_2_CO_3_ and 140 μL of distilled water were added. Finally, the mixture was allowed to stand for 30 min, and the absorbance was determined at 760 nm. The results were expressed in milligrams of gallic acid equivalents per gram of the dry sample (mg GAE/g).

#### Statistic Analysis

A statistical analysis was performed, where the triplicate measurements of the data obtained in each test or determination were evaluated, and the mean ± standard deviation was expressed. A Tukey comparison of means was performed at *p* ≤ 0.05 to determine significant differences between treatments by analysis of variance (ANOVA) with InfoStat software.

## Conclusions

The extraction of betalains by ultrasonication, followed by an ultrafiltration process, will result in an extract with excellent antioxidant properties. The coaxial electrospray technique was useful for obtaining gelatin–betalain nanoparticles, maintaining the antioxidant properties of betalains when encapsulated; due to the origin of its components and given that the use of toxic solvents is not necessary for the technique, the material obtained can be considered food grade.

It is concluded that the best parameters for obtaining gelatin–betalain nanoparticles by coaxial electrospraying are a betalain concentration of 3% w/v in the core and 10% w/v gelatin as a cover, flow rates of the polymer solution of 50 μL/h for betalains and 0.1 mL/h for gelatin, a voltage of 15 kV, and a distance between the needle and the collector plate of 10 cm. Obtaining nanoparticles with which they were finally the ones that showed an adequate spherical morphology, with a tendency to monodispersity, without fiber residues, in addition to thermogravimetric techniques, it was revealed that this material mainly protected the betalains, giving them greater stability against high temperatures. Therefore, it is possible to use these gelatin nanoparticles as encapsulants for the betalain bioactive compound. Through the spectra obtained with the FTIR technique, it was verified that gelatin and betalain interacted with each other. Likewise, by the ABTS, DPPH, and total phenols methods, a high antioxidant capacity was presented in the extract and was maintained in the coaxial nanoparticles due to the favorable interaction, which confirmed that the betalains retained their antioxidant activity, that is, no degradation occurred. The result of this research serves as a contribution to the research on core–shell materials, demonstrating that gelatin and betalains are potential materials to be applied as coatings in functional foods.
